# Distinctive and common features of moderate aplastic anaemia

**DOI:** 10.1111/bjh.16460

**Published:** 2020-01-31

**Authors:** Bhumika J. Patel, Shimoli V. Barot, Teodora Kuzmanovic, Cassandra Kerr, Bartlomiej P. Przychodzen, Swapna Thota, Sarah Lee, Saurabh Patel, Tomas Radivoyevitch, Alan Lichtin, Anjali Advani, Matt Kalaycio, Mikkael A. Sekeres, Hetty E. Carraway, Jaroslaw P. Maciejewski

**Affiliations:** 1 Department of Translational Hematology and Oncology Research, Taussig Cancer Institute, Cleveland Clinic; 2 Leukemia Program, Department of Hematology and Medical Oncology, Taussig Cancer Institute, Cleveland Clinic, Cleveland, OH; 3 Department of Leukemia, Roswell Park Comprehensive Cancer Center, University of Buffalo, Buffalo, NY; 4 Department of Quantitative Health Sciences, Lerner Research Institute, Cleveland Clinic, Cleveland, OH, USA

**Keywords:** moderate aplastic anaemia, myelodysplastic syndrome, acute myeloid leukaemia, molecular mutation, clinical outcomes

## Abstract

The therapy algorithm for severe aplastic anaemia (sAA) is established but moderate AA (mAA), which likely reflects a more diverse pathogenic mechanism, often represents a treatment/management conundrum. A cohort of AA patients (*n* = 325) was queried for those with non-severe disease using stringent criteria including bone marrow hypocellularity and chronic persistence of moderately depressed blood counts. As a result, we have identified and analyzed pathological and clinical features in 85 mAA patients. Progression to sAA and direct clonal evolution (paroxysmal nocturnal haemoglobinuria/acute myeloid leukaemia; PNH/AML) occurred in 16%, 11% and 1% of mAA cases respectively. Of the mAA patients who received immunosuppressive therapy, 67% responded irrespective of time of initiation of therapy while conservatively managed patients showed no spontaneous remissions. Genomic analysis of mAA identified evidence of clonal haematopoiesis with both persisting and remitting patterns at low allelic frequencies; with more pronounced mutational burden in sAA. Most of the mAA patients have autoimmune pathogenesis similar to those with sAA, but mAA contains a mix of patients with diverse aetiologies. Although progression rates differed between mAA and sAA (*P* = 0·003), cumulative incidences of mortalities were only marginally different (*P* = 0·095). Our results provide guidance for diagnosis/management of mAA, a condition for which no current standard of care is established.

Most of the idiopathic aplastic anaemias (AA) result from immune-mediated destruction of the haematopoietic progenitor stem cells (HPSC) leading to varying degrees of cytopenia (Young & Maciejewski, 1997). For decades, the clinical Camitta criteria have been used to categorize acquired AA according to the degree of myelosuppression and cellularity of the bone marrow, either as severe (sAA) or very severe (vsAA), with moderate (mAA) defined as not meeting the definition of the former two, and exclusion of other entities such as congenital bone marrow failure (BMF) states, hypocellular myelodysplastic syndromes (MDS) or secondary causes of marrow aplasia (Camitta et al., 1976; Bacigalupo et al., 1988; Young et al., 2006; Afable et al., 2011a,b; Nazha et al., 2015).

Once the general diagnosis of AA is established, sAA and vsAA are easily discerned by the severity of myelosuppression. In contrast, AA patients not fulfilling the severity criteria often constitute a diagnostic and management challenge. Excluding other aetiologies of BMF, the initially mildly depressed counts may represent an early stage of sAA and the diagnosis of true mAA may require a longer clinical follow-up. To avoid ambiguities, we propose to distinguish evolving sAA with moderate cytopenias from true mAA, characterized by chronic moderately depressed blood counts without clinical evidence of progression.

Proper diagnosis of pancytopenia in the context of an ‘empty bone marrow’ is clinically paramount. For instance, hypocellular MDS with moderate cytopenias can be treated with hypomethylating agents or, like sAA, with immunosuppressive therapy (IST), whereas congenital BMF may be IST-refractory. Even when the diagnosis of mAA is established, the management of transfusion-independent patients is not clear. While monitoring is appropriate for some patients, there are others who should receive IST to prevent progressive destruction of the HPSC. Despite all efforts to establish underlying pathogenesis, mAA may harbour clinical heterogeneity, making assessment of therapy responses difficult.

Given the ambiguities in real time and implications for management, insights into the pathogenesis, natural history and proper management can be gained through retrospective analysis of mAA. Consequently having collected a large cohort of mAA patients, we performed clinical and molecular analyses to identify characteristics that could accurately distinguish and diagnose true mAA from other conditions, as well as provide anecdotal evidence for clinical management.

## Patients and Methods

### Patients

We retrospectively analyzed a cohort of 325 patients with the diagnosis of AA from 1998 to 2018 who were referred for evaluation at our institution, of which 88 had mAA, 230 had sAA, and seven had vsAA (Table I; Table SI) based on the established criteria (Camitta et al., 1976; Bacigalupo et al., 1988; Marsh et al., 1999; Maciejewski et al., 2003). Informed consent was collected in accordance to the Declaration of Helsinki and Institutional Review Board policies.

For the purpose of this study, mAA was defined by bone marrow cellularity <25% with one or more of the following clinically meaningful cytopenias: absolute neutrophil count (ANC) <1·5 × 10^9^/l, absolute reticulocyte count (ARC) <60 × 10^9^/l or platelet <150 × 10^9^/l and not meeting the criteria for sAA/vsAA. Diepoxybutane (DEB) testing was performed for all children and young adult patients suspected for Fanconi anaemia (FA) and other forms of congenital BMF were excluded by appropriate testing indicated by the clinical scenario. We evaluated patients for evolution to sAA/PNH (paroxysmal nocturnal haemoglobinuria)/MDS (myelodysplastic syndrome)/AML (acute myeloid leukaemia) (at three and six months and after an extended average of 72 months (range 8–278). Of the 88 patients, three were excluded from the mAA cohort due to progression to sAA (confirmed by bone marrow biopsy and degree of cytopenias) or clinically significant haemolytic PNH requiring treatment within the three-month period. Two patients progressed to sAA/PNH by the six-month mark and the rest progressed during extended follow-up. In addition to marrow hypocellularity, the absence of other causes of lineage-restricted cytopenia, including pure red cell aplasia, MDS, ‘pure’ PNH without AA, idiopathic/immune thrombocytopenic purpura (ITP), amegakaryocytic thrombocytopenia, severe congenital neutropenia, large granular lymphocytic leukemia (LGL) and non-Hodgkin lymphoma has been asserted by appropriate clinical testing and bone marrow morphology (Barrett et al., 2000; Jaffe et al., 2001; Bennett & Orazi, 2009). DNA sequencing was available for 62 of the 85 mAA patients (73%), including both somatic and germline mutations in BMF disorders and myeloid malignancies (Table SII). Likely pathogenic germline variants (designated as Tier 1) in heterozygous configuration were found in 11% of patients (Table SIII).

The mAA cohort was established based on at least two measurements of complete blood counts obtained at two different time points and meeting the established criteria (Table SI). Clinical data were obtained from the time of diagnosis, throughout the clinical course and in conjunction with their respective DNA sequencing sample collection. The cumulative incidence of progression and mortality was calculated from the time of diagnosis to last follow-up, which was defined based on the patient’s most recent available data at our institution. Responses were modified based on the established criteria used in prior studies for sAA and mAA (Rosenfeld et al., 1995; Sloand et al., 2010). Complete haematological response (CR) was defined as normalization of blood counts at three and six months in sAA, while normalization of two serial measurements at least one month apart was indicative of CR in mAA. Partial response (PR) and no response (NR) were classified based on previous studies (Tables SIV and SV) (Rosenfeld et al., 1995; Sloand et al., 2010). For the purpose of our study, cytopenias were classified as mild, moderate, and severe using the following ranges: haemoglobin (>100; 80–100; <80 g/l), ANC (>1·0; 0·5–1·0; <0·5 × 10^9^/l) and platelets (>100; 50–100; <50 × 10^9^/l), respectively.

### Cytogenetic analysis

Cytogenetic analysis was performed on marrow aspirates and/or peripheral blood. Twenty metaphase spreads were examined per patient (mAA: *n* = 70; sAA/vsAA *n* = 177) with no growth/non-informative cytogenetics in *n* = 15 mAA and *n* = 60 sAA. Chromosome preparations were G-banded using trypsin and Giemsa (GTG) and karyotypes were described according to the International System for Human Cytogenetic Nomenclature (Sugimoto et al., 2012). Single nucleotide polymorphism (SNP) array-based karyotyping was available for 32 patients in the mAA cohort (Data S1).

### Nextera custom capture and targeted sequencing

Capture of a custom gene panel of 186 somatic and congenital genes found in BMF disorders and myeloid neoplasms was carried out using the Nextera Custom Enrichment kit following standard manufacturer protocols (Illumina Inc., San Diego, CA, USA), and deep sequencing was performed using the HiSeq2000 platform (Illumina) on the patient’s bone marrow aspirates and/or peripheral blood samples (Table SII). Tier 1 variants were defined based on criteria using an in-house implemented pipeline as previously described (Data S1, Figures S1 and S2) (Patel et al., 2017; Hirsch et al., 2018; Przychodzen et al., 2018).

### Statistical analysis

The competing-risk method of Fine and Gray as implemented in the R package cmprisk was used to generate and compare cumulative incidence curves (Gray, 1988; Fine & Gray, 1999). To compare frequencies of mutations between groups the Fisher exact test was used. Two-sided *P* values ≤0·05 were considered statistically significant. The statistical environment R was used for all computations (R Foundation for Statistical Computing, Vienna, Austria, www.r-project.org).

## Results

### Diagnostic delineation of mAA

Of the 325 patients with AA, 88 met the criteria (Camitta et al., 1976; Bacigalupo et al., 1988; Marsh et al., 1999; Maciejewski et al., 2003) for the diagnosis of mAA and 237 those for sAA/vsAA by the degree of myelosuppression and bone marrow cellularity. However, among the mAA cohort, two progressed to sAA within a short span of less than three months, while one was found to have clinically significant haemolytic PNH, prompting reclassification and exclusion from the study. The remaining 85 patients were classified as mAA following full initial evaluation and constituted the studied cohort. Per definition, in addition to marrow hypocellularity, the blood counts were moderately depressed as follows: all three lineages affected in 40/85 cases, 2/3 lineages in 30 cases, with thrombocytopenia/anaemia as the most common combination, and initial unilineage involvement in 15 cases, with all cases eventually progressing to bi/pancytopenia over an extended follow-up of ~10 months (range 4–23) prior to initiation of therapy (Table SVI). The median marrow cellularity was 20% (range 5–25%), and 29/85 patients were transfusion-dependent, defined as requiring at least two units of red blood cells or five units of platelets for a period of two months (Sloand et al., 2010).

**Fig 1.**
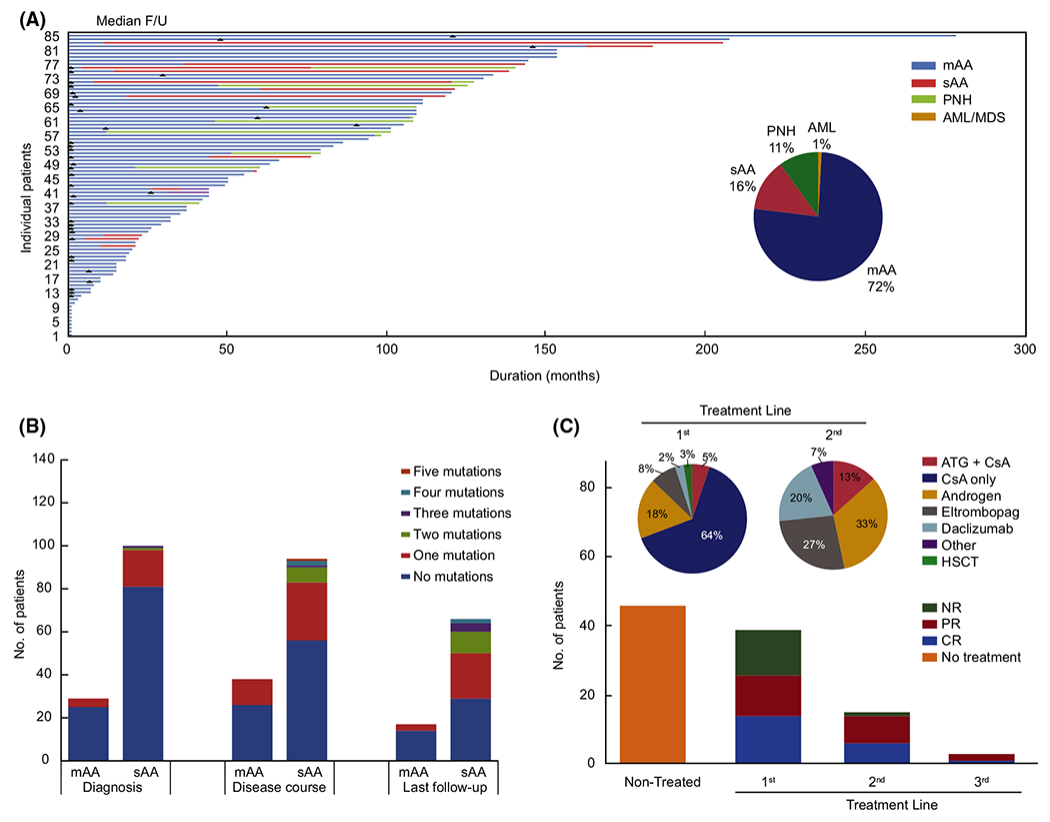
Clinical course, treatments and response to therapy. (A) The horizontal bar graph represents individual pts with moderate aplastic anaemia (mAA) with their respective clinical course (*n* = 85) and the start of mAA therapy is depicted by the black triangle. The pie chart depicts the proportions of pts with mAA and those that transformed to severe aplastic anaemia (sAA), PNH and AML. (B) Clonal dynamics of mAA *versus* sAA: at diagnosis, during clinical course, and last follow-up prior to progression. (C) The pie diagrams indicate 1st (left) and 2nd (right) lines of treatment. The bar graphs depict the individual responses to therapy. Supportive care not included (transfusions, growth factors, and antibiotics). ATG, anti-thymocyte globulin; CsA, ciclosporin; HSCT, haematopoietic stem cell transplant; NR, no response; PR, partial response; CR, complete response.

The median age for the mAA cohort was 43 years (range: 6–88; Table I) with a median follow-up of 49 months (range: 1–278). The metaphase karyotype at diagnosis was informative for 70/85 patients, 69 of whom had a normal karyotype with one having trisomy 15. SNP was available for 32/85 patients, of which seven had evidence of uniparental disomy without a specific clinical correlation. At the last follow-up during the clinical course of mAA, cytogenetic analysis was performed in 28/70 patients of which normal karyotype was noted in 24 patients. Four patients progressed from mAA to sAA to MDS; one patient acquired additional chromosome 6 in addition to their prior chromosomal abnormality of trisomy 15 and three patients acquired monosomy-7 at the time of MDS progression. Clinically, 14 mAA patients (16%) (Fig 1A) progressed to sAA, with a median time to progression of 31 months (4–162 months). One patient with mAA and two who progressed to sAA went on to receive a haematopoietic stem cell (HSC) transplant. Among the 237 patients with sAA at initial diagnosis, 53 (22%) received a transplant in the up-front setting or during their clinical course for sAA and four (2%) went to transplant due to progression to MDS/AML.

### Comparative study of clinical, biological and genomic features of mAA versus sAA

While the degree of myelosuppression alone has been used to classify mAA *versus* sAA, there may be other unique clinical and genomic features to distinguish the two entities. At diagnosis, 29/85 and 201/237 patients in the mAA and sAA cohorts, respectively, were transfusion-dependent (34% vs. 85%, *P* = 0·0001; Table I). Of the transfusion-dependent patients in the mAA cohort, 3% (1/29) had unilineage involvement, 28% (8/29) had bilineage involvement and 69% (20/29) had pancytopenia. Of these, 59% (17/29) received only platelets, 34% (10/29) red blood cells (RBCs), and 7% (2/29) received both platelets and RBC transfusions. In comparison, among those who were transfusion-independent, 25% (14/56) had unilineage involvement, 39% (22/56) had bilineage involvement and 36% (20/56) had pancytopenia. The most common cell lines to be affected were platelets (77/85, 91%) followed by erythrocytes (69/85, 81%) and neutrophils (49/85, 58%). In contrast, in the sAA cohort pancytopenia was found in 54% (127/237), and two lines were involved in 46% (110/237) (Table SVI).

We additionally subdivided the patients based on the severity of cytopenias as defined in the *Patients and methods* section. In 71% (10/14) with mAA who eventually progressed to sAA, patients had severe cytopenias and were transfusion-dependent. In contrast, only 10/71 patients (14%) who did not progress to sAA had similar severity of myelosuppression and transfusion dependency (*P* = 0·0001; Fig 1A). The two clinical risk factors that predicted progression in most cases were severity of cytopenias and transfusion dependency. PNH clones >1% were present in 26% (22/85) mAA vs. 23% (54/237, *P* = 0·55) sAA/vsAA patients, with the average glycosylphosphatidyl-inositol (GPI) anchor-deficient granulocyte clone 28% (range 2–63%) in mAA vs. 36% (4–76%) in sAA at diagnosis. In the mAA group nine patients (11%) progressed to clinically significant haemolytic PNH of whom eight had a PNH clone present at diagnosis, and one patient acquired a PNH clone during the course of disease (Fig 1A). In the sAA group, 22 patients (9%) progressed to haemolytic PNH with 12 patients having a clone present at diagnosis and 10 patients acquiring a PNH clone over the course of their disease.

Mutations were analyzed for 73% (62/85) mAA patients on a panel consisting of genes known to be somatic and germline mutations in BMF disorders and myeloid malignancies and 11 had serial samples (Table SII). Tier 1 germline alterations with a likely pathogenic impact in heterozygous configuration were found in 11% (7/62); however, none of the patients were found to carry autosomal dominant alterations or were bi-allelic carriers (Table SIII). Somatic hits were detected in four patients in the mAA cohort at diagnosis with a sole mutation in one of the following: *RUNX1, ASXL1, PIGA* and *PTPN11*. Comparably, 19% sAA/vsAA cases had at least one mutation at diagnosis (*P* = 0·59) with most frequent mutations in *DNMT3A*, *PIGA*, *SRSF2* and *CEBPA*. Upon combined genomic analyses during the disease course, 32% of mAA vs. 40% of sAA/vsAA showed no difference in the average number of mutations. However, at last follow-up, more mutations were found in sAA/vsAA patients, 56% vs. 18% in mAA (*P* = 0·006; Fig 1B; Table SVII). Data suggest that the proportion of genomic hits corresponded to the unstable haematopoietic clones. For instance, a transient *RUNX1* mutant clone was found in one patient who did not have evidence of clonal evolution. In another case an *ASXL1* mutant clone persisted to eventually progress to MDS with monosomy 7, where the clone expanded (3→ 19%). A similar course was observed for four patients with a *PIGA* mutation, whose clone expanded resulting in a progression to clinically relevant haemolytic PNH requiring treatment, which was also confirmed by flow cytometry with a lower limit of detection of 0·01% for PNH-type cells. In addition, four patients had clinically relevant PNH by flow cytometry, but no correlative genomic sequencing data. The next-generation sequencing data from the last follow-up revealed that 2/11 patients acquired a single mutation in *ETV6* (variant of uncertain significance [VUS]; variable allele frequency [VAF]: 48%) and *ASXL1* (VAF: 15%).

### Therapy and outcomes

About half of the mAA patients (54% [46/85]) did not receive any active treatment *versus* 3% (8/237) sAA patients who received supportive therapy alone. Treatment modalities for mAA and sAA patients (Fig 1C) were similar, including antithymocyte globulin (ATG; equine or rabbit), androgens, immunosuppressive therapy (IST; calcineurin inhibitors, daculizumab or mycophenolate mofetil), eltrombopag, and HSC transplant (Table SVIII). Factors influencing the choice of treatment modality were individually determined based on clinical acuity, severity of cytopenias, transfusion dependency and other clinical factors. For instance, single-agent IST was usually applied to mAA with a PNH clone without severe cytopenias, single-agent danazol may have been used in patients with mild renal impairment and moderate cytopenias, while patients with thrombocytopenia as a leading symptom were likely to be treated with eltrombopag. As expected, ATG plus ciclosporin (CsA) was administered less often in mAA 2/39 (5%) than in sAA 164/229 (72%; *P* = 0·0001) due to the clinical phenotype in the up-front setting. In comparison to sAA, androgen therapy 7/39 (18%) vs. 7/229 (3%; *P* = 0·0013), IST 26/39 (67%) vs. 33/229 (14%; *P* = 0·0001), eltrombopag 3/39 (8%) vs. 5/229 (2%; *P* = 0·095) and HSC transplant 1/39 (3%) vs. 20/229 (9%; *P* = 0·33) were among first-line therapies (Table SIX).

In comparison to the 95/237 (40%) sAA/vsAA patients, 24/85 (28%) of the mAA patients required one line of treatment (*P* = 0·0002), 12/85 (14%) vs. 66/237 (28%) required two (*P* = 0·0119) whereas 3/85 (4%) vs. 39/237 (17%) required three lines of treatment (*P* = 0·0013). In sAA, it was noted that 12% (29/237) had refractory disease whom required four or more lines of therapy, whereas most mAA patients with stable or mildly progressive disease required only one or two lines of therapy (Table I).

Responses to first-line therapy were evaluated based on established criteria (Rosenfeld et al., 1995; Sloand et al., 2010). Therefore mAA patients who required treatment (*n* = 39) were subclassified based on their transfusion status. CR/PR rate in transfusion-dependent patients was 59% (16/27) and 75% (9/12) in transfusion-independent patients. Of these patients 42% (11/27) were refractory to treatment vs. 25% (3/12; *P* = 0·50). Time to response in mAA, where first-line therapy was IST, was approximately 12 months in patients who achieved a CR/PR. Evaluation of response to intensive IST with ATG plus CsA as first-line therapy was evaluated for 2/85 with mAA vs. 164/237 with sAA, with a median time to response of three *versus* eight months (*P* = 0·0001). We also compared ATG plus CsA responses in sAA with antecedent mAA (*n* = 13) *versus* ‘primary’ sAA (*n* = 164); of the mAA (14/85) that eventually evolved to sAA, 62% (8/13) achieved a CR, 23% (3/13) PR, 15% (2/13) NR with one patient censored due HSC transplant. Forty-six per cent (6/13) of these patients received prior therapies for mAA before evolving to sAA of which 83% (5/6) had a GPI-deficient granulocyte clone. In contrast to those with sAA at diagnosis, responses to ATG plus CsA were 34% (55/164; *P* = 0·07) CR, 27% (45/164; *P* = 1·00) PR, 39% (64/164; *P* = 0·14) NR with 20 patients receiving HSC transplant up front. There was no difference in response to therapy in those who evolved to sAA from mAA.

### Clinical and survival outcomes

**Fig 2.**
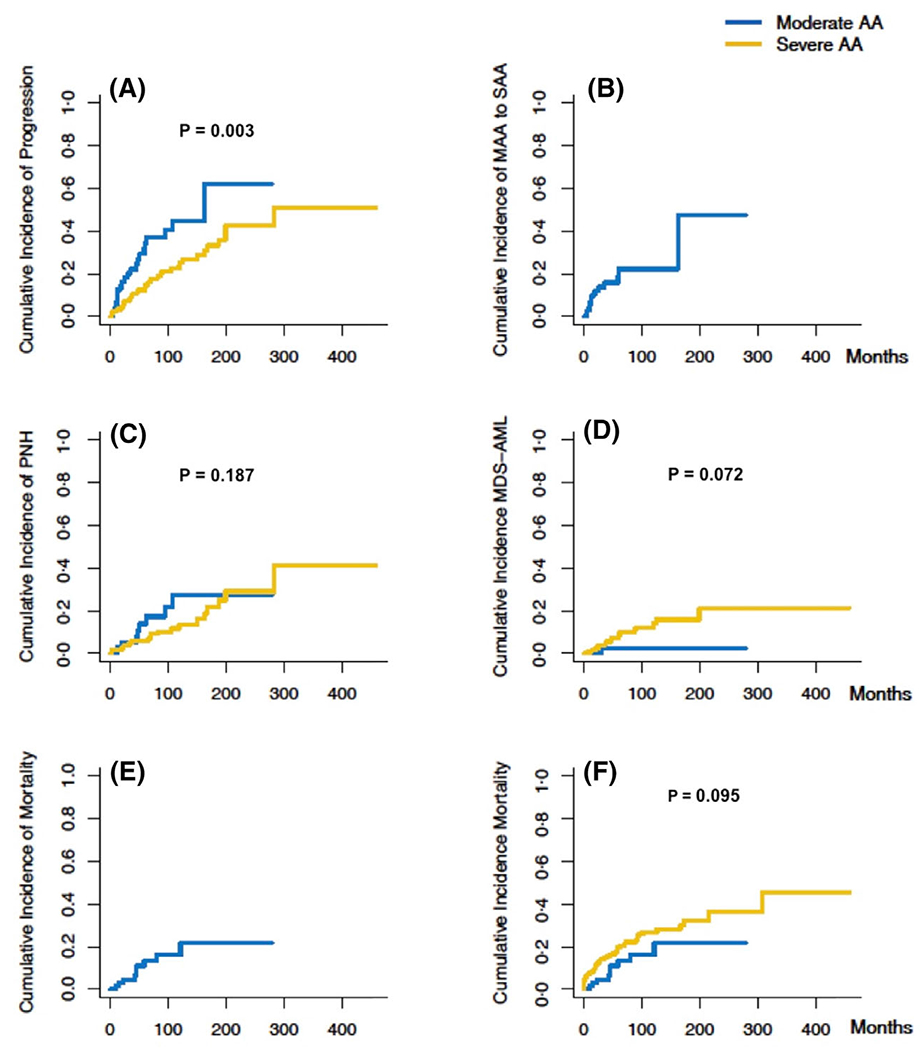
Cumulative incidence of progression and mortality in moderate (mAA) and severe (sAA) aplastic anaemia. (A) Cumulative incidence (CI) of progression in mAA and sAA. (B) CI of mAA to sAA. (C) CI of paroxysmal nocturnal haemoglobinuria (PNH) between mAA and sAA. (D) CI of MDS/AML in mAA and sAA. (E) CI of mortality in the mAA patients (non-progressors *n* = 61) (F) CI of mortality in mAA and sAA.

Across the entire mAA cohort, 16% of patients (14/85) progressed to sAA, 11% (9/85) to clinically significant PNH requiring initiation of eculizumab therapy and 1% (1/85) progressed AML. In contrast, in sAA 9% (22/237) of patients progressed to clinically significant PNH requiring eculizumab therapy and 9% (22/237) progressed to AML or MDS. The cumulative incidence (CI) of progression at five years showed a higher proportion of patients progressing in mAA *versus* sAA, 32% vs. 13% (*P* = 0·003), with the limitation of a shorter follow-up duration for the mAA cohort (Fig 2A). The mean time to progression from mAA to sAA was 41 months with a five-year CI of 19% (Fig 2B). Subanalysis of progression to PNH and MDS/AML noted no significant difference between the two cohorts (*P* = 0·187; *P* = 0·072) respectively (Fig 2C, D). The five-year incidence of mortality was 19% in the mAA patients (non-progressors *n* = 61) and there was no difference between mAA and sAA (*P* = 0·095) (Fig 2E,F). The most common causes of death in the mAA cohort (*n* = 9) included progression to AML, uncontrolled infection, cardiac arrest or complications related to medical comorbidities.

## Discussion

We set out to distinguish unique characteristics to support the management algorithm for moderate idiopathic AA, which is yet to be established. Definition of sAA has been defined by the Camitta criteria (Camitta et al., 1976) but patients not meeting the criteria for severe or very severe have often been referred to as moderate. For clarity of the definition of mAA and exclusion of ‘diagnostic noise’ we evaluated our cohort at different time points to assess for clinical progression. While the approach to patients with sAA has been widely standardized and non-controversial, medical management of those with seemingly idiopathic mAA varies widely. One could stipulate that early treatment would protect the HPSC pool from exhaustion, while conversely, mAA may be the end result of stem cell damage and further IST may not be effective. We show in this single centre analysis that mAA stringently distinguished from similar, but distinct, diagnoses constituted 26% of all AA patients. With our retrospective analysis we were able to identify clinical outcomes and risk factors for progression to PNH or MDS/AML, which appear to be similar to sAA. Treatment for mAA remains undefined by rigorous studies or consensus panels. Our treatment algorithm was formulated and individually adjusted based on clinical acuity, severity of myelosuppression, and transfusion dependency, which were identified as risk factors of progression based on our analysis.

Among 14 patients who progressed to sAA, four had moderate counts and were not transfusion-dependent, suggesting that disease severity is not always associated with a greater risk of sAA progression. Eight patients with severe cytopenias and transfusion dependency progressed on average 29 months despite treatment with non-ATG-based therapy. Six patients with moderate counts and transfusion independence progressed on average 34 months. These observations illustrate the importance of early treatment with ATG plus CsA to prevent burnout of the bone marrow from chronic attack by cytotoxic T cells as other studies have found (Marsh et al., 1999; Jiang et al., 2009; Nishio et al., 2009; Nishikawa et al., 2017). However, patients with a long course of mAA were still able to respond to late IST treatment, an observation that may contradict the theory that prolonged disease process depletes HPSC (Khatib et al., 1994; Brock et al., 2013). Similar to sAA, mAA may also be a precursor to evolving haemolytic PNH, as noted with a subset of mAA patients who had mild cytopenias and were transfusion-independent. Our findings suggest that chronic mAA may be its own unique biological entity with an indolent course. Indeed 62% (37/60) of the patients required no treatment and were monitored periodically without progression. Of the mAA patients (*n* = 23) who did receive treatment, most were symptomatic despite moderate blood count depression or were severely affected in a single lineage; e.g. required transfusions. Conversely, those who progress suggest a continuum of disease and clonal evolution.

Between our mAA and sAA cohorts there was no significant difference in age. However evidence of clonal haematopoiesis was noted in patients as young as 20 years old, but was more common in the older population as reported in other studies (Genovese et al., 2014; Jaiswal et al., 2014; Ogawa, 2016). Early genomic studies have shown that SNP array karyotyping may increase the detection rate of chromosomal lesions especially microdeletions and uniparental disomy. Identification of such cryptic lesions could change the diagnoses to hypocellular MDS (Afable et al., 2011b). Recent genomic studies in sAA have discovered clonal abnormalities in a disorder previously thought of as a non-clonal disorder, which have detected somatic hits of which some were prognostically favourable or neutral while others impacted outcomes (Kulasekararaj et al., 2014; Shen et al., 2014; Babushok et al., 2015; Yoshizato et al., 2015; Young & Ogawa, 2015; Ogawa, 2016; Negoro et al., 2017). In serial studies, mutations such as *CBL*, *SETBP1* and *RUNX1* were shown to predict the risk of MDS evolution (Negoro et al., 2017). Within our retrospective study a few molecular lesions were found in mAA, and the mutational burden was more significant for sAA, illustrating the chronic course of the disease and also predicting for disease evolution. In addition, carriers of germline alterations may be a reflection of genetic predisposition with low or incomplete penetrance. The severity of cytopenias and the chronicity of the disease may drive clonal selection and acquisition of additional clones by which unrelated diagnoses might be more easily confused with mAA than with sAA. Future in-depth molecular studies in mAA will further clarify the molecular profile for this unique entity and will discern it from clonal haematopoiesis.

**Fig 3.**
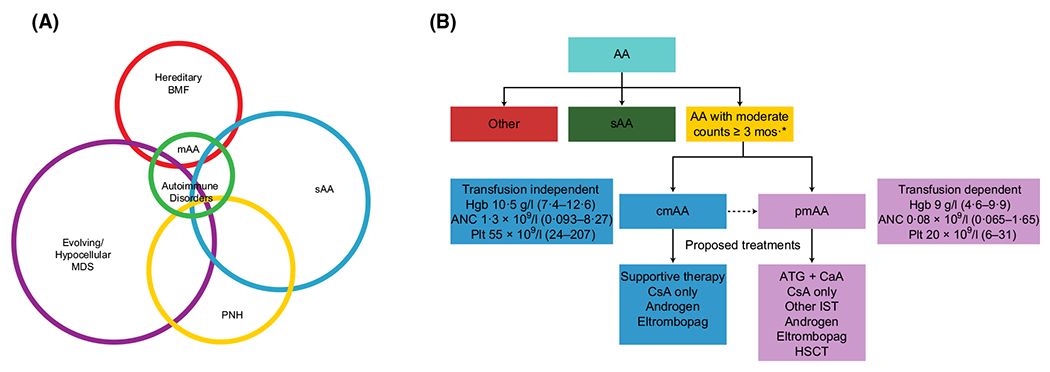
Diagnosis and treatment algorithm. (A) Pathogenic and diagnostic overlap of mAA. (B) Treatment algorithm for AA with moderate counts: chronic moderate aplastic anaemia (cmAA) without clinical progression and progressive moderate aplastic anaemia (pmAA) with clinical presentation similar to that of sAA. BMF, bone marrow failure; Hgb, haemoglobin; ANC, absolute neutrophil count.

In sum, the results of our retrospective analysis of clinical diagnostic steps, management and outcome data for mAA provides a guide for an orphan diagnosis for which a current standard of care is poorly defined with limited studies (Marsh et al., 1999; Maciejewski et al., 2003; Sloand et al., 2010; Nishikawa et al., 2017). Several retrospective studies have been performed to identify risk factors for progression; however, specific associations have been limited to low absolute reticulocyte, neutrophil and platelet counts as predictors of progression (Howard et al., 2004; Kwon et al., 2010; Wang et al., 2013). With evaluation at different time intervals of ≥3 months and identifying factors of progression, we propose that mAA can either remain a chronic entity or have risk factors for early progression requiring early and aggressive treatment as one would for sAA. Due to the retrospective nature of the analysis, the lack of a validation cohort, limited sample size, duration of follow-up, molecular data, and overlap of other BMF disorders, there may be ascertainment and recruitment bias. In addition, further serial genomic sequencing of a larger cohort of patients with mAA will help characterize this unique entity. With the arbitrary classification and lack of established guidelines for management of mAA, we have summarized the approach to diagnosis and treatment derived from our clinical experience in Fig 3A,B. Limitations of the study highlight the need for randomized control trials and establishment of a consensus guideline.

## Supplementary Material

supporting information 1 **Data S1.** Supplemental methods.
**Fig S1.** Bioanalytic algorithm for somatic variants.
**Fig S2.** Bioanalytic algorithm for germline variants.

supporting information 2 **Table SI.** Classification of aplastic anaemia.
**Table SII.** Panel of tested somatic and germline mutations.
**Table SIII.** Tier 1 germline variants.
**Table SIV.** Response criteria for sAA.
**Table SV.** Response criteria for mAA.
**Table SVI.** Subclassification of cytopenias in mAA and sAA patients.
**Table SVII.** Mutational profile of mAA.
**Table SVIII.** Modalities of treatment received for mAA.
**Table SIX.** Responses to therapy.

## Figures and Tables

**Table I.** Clinical characteristics of patients with mAA (*n* = 85) and sAA/vsAA (*n* = 237).

	mAA	sAA/vsAA
Demographics		
Age, years, median (range)	43 (6–88)	48 (3–89)
Sex, male/female	38/47	123/114
Median follow-up		
Since diagnosis, months, (range)	49 (1–278)	58 (1–642)
Laboratory data at diagnosis		
Haemoglobin, median (g/l)	100	90*
Neutrophils, median (× 10^9^/l)	1·35	0·65*
Platelets, median (× 10^9^/l)	47	17*
Absolute reticulocyte count, median (M/l)	0·055 × 10^6^	0·025 × 10^6,^*
PNH clone present, yes/no	22/63	54/183
PNH WBC clone, average (%)	28%	36%
Lines of treatment (tx), (%)		
Transfusion-dependent	34%	85%**
Supportive care	54%	3%
1 line of tx	28%	40%
2 lines of tx	14%	28%
3 lines of tx	4%	17%
≥4 lines of tx	0%	12%

mAA, moderate aplastic anaemia; sAA, severe aplastic anaemia; vsAA, very severe aplastic anaemia; PNH, paroxysmal nocturnal haemoglobinuria; WBC white blood cell; Hgb, haemoglobin; ANC, absolute neutrophil count; cmAA, chronic moderate aplastic anaemia; pmAA, progressive moderate aplastic anaemia; ATG, anti-thymocyte globulin; CsA, ciclosporin; IST, immunosuppressive therapy; HSCT, haematopoetic stem cell therapy.

* *P* = NS;

** *P* = 0·0001.

## Key Points

- mAA is either a precursor of sAA/PNH and on rare occasion MDS/AML or a distinct chronic, almost non-progressive entity characterized by cytopenias, transfusions and delayed time to evolution.
- Retrospective analyses provide an evidence-based algorithm for mAA management and treatment.
