# Supplementary material for: Distinctive and common features of moderate aplastic anaemia
Source: Br J Haematol. Author manuscript; Available in PMC 2021 Aug 5. (PMC8340733; doi:10.1111/bjh.16460)
Supplement: supporting information 1 — Data S1. Supplemental methods. Fig S1. Bioanalytic algorithm for somatic variants. Fig S2. Bioanalytic algorithm for germline variants. [file NIHMS1719035-supplement-supporting_information_1.docx]

**Supplementary appendix**

**Content**

**1. Supplemental Methods**

1.1 Single nucleotide polymorphism-array analysis

1.2 Targeted sequencing pipeline

**2. Supplemental Tables**

Supplemental Table 1. Classification of aplastic anemia

Supplemental Table 2. Panel of tested somatic and germline mutations

Supplemental Table 3. Tier 1 germline variants

Supplemental Table 4. Response criteria for sAA

Supplemental Table 5. Response criteria for mAA

Supplemental Table 6. Subclassification of cytopenias in mAA and sAA patients

Supplemental Table 7. Mutational profile of mAA

Supplemental Table 8. Modalities of treatment received for mAA

Supplemental Table 9. Responses to therapy

**3. Supplemental Figures**

Supplemental Figure 1. Bioanalytic algorithm for somatic variants

Supplemental Figure 2. Bioanalytic algorithm for germline variants

**1. Supplemental Methods**

**1.1 Single nucleotide polymorphism-array analysis**

SNP-array karyotyping for confirming metaphase cytogenetics and detecting copy number normal loss of heterozygosity was performed in 32/85 patients as previously described.(Makishima*, et al* 2011, Makishima*, et al* 2012) Briefly, Affymetrix 250 K and 6.0 (Affymetrix, Santa Clara, CA, USA) SNP-arrays were used to evaluate copy number and loss of heterozygosity. Using our internal and publicly available databases (see URLs), the screening algorithm validated each lesion as somatic vs germ line.(Huh*, et al* 2010, Tiu*, et al* 2009) Non-somatic lesions were excluded from further analysis. Affected genomic positions in each lesion were visualized and extracted by CNAG (v3.0, Tokyo, Japan) or the Genotyping Console (Affymetrix) software.

**1.2 Multi-amplicon deep sequencing**

Multi-amplicon deep sequencing (TruSeq; Illumina) was performed for 64 gene targets, according to the manufacturer’s instructions (Illumina) [**Supplementary Figure 1 & 2 and Supplementary Table 2**]. Customized primer sets were designed to amplify exons of target genes. The libraries were subjected to deep sequencing on MiSeq (Illumina) sequencers according to the standard protocol. Subsequent validation and confirmatory sequencing are described as below [**Supplementary** **Figure 1 & 2**].

**Nextera custom capture, targeted sequencing**

Capture of a custom panel of 186 genes was carried out using the Nextera Custom Enrichment kit (protocol:http://support.illumina.com/content/dam/illuminasupport/documents/documentation/chemistry_documentation/samplepreps_nextera/nexteradna/nextera-dna-library-prep-reference-guide-15027987-01.pdf) following standard manufacturer protocols (Illumina), and sequencing was performed using a HiSeq2000 (Illumina). Data analysis used an in-house implemented pipeline. Paired-end reads (2x100bp) were aligned (BWA v0.91 to a reference genome GRCh37/hg19)(Li and Durbin 2010) calling and filtering were done using GATK4(Van der Auwera*, et al* 2013) and variant annotation carried out with Annovar.(Wang*, et al* 2010) The minimum depth of 10 reads and 4 positive mutant reads were required for a variant to be considered for further processing. Variants reported in public databases (Lek*, et al* 2016) with allele frequency above 0.001 were removed as common, inherited variation. CADD score (>=15.0) was used to predict the functional impact of variants, and retain missense/nonsense/frameshift changes.(Kircher*, et al* 2014) Variants were prioritized on the basis of the functional relevance of variant/genes (known somatic variants, novel LOF/frameshift/stopgain/splice/high CADD index) [**Supplementary Figure 1**]. Tier 1 germline variants with likely pathogenic impact were detected as described in [**Supplementary Figure 2**]. Refer to https://support.illumina.com/content/dam/illumina-support/documents/documentation/chemistrydocumentation/samplesprepsnextera/nexteradna/nextera-dna-library-prep-reference-guide-15027987-01.pdf.

**2. Supplemental Tables**

**
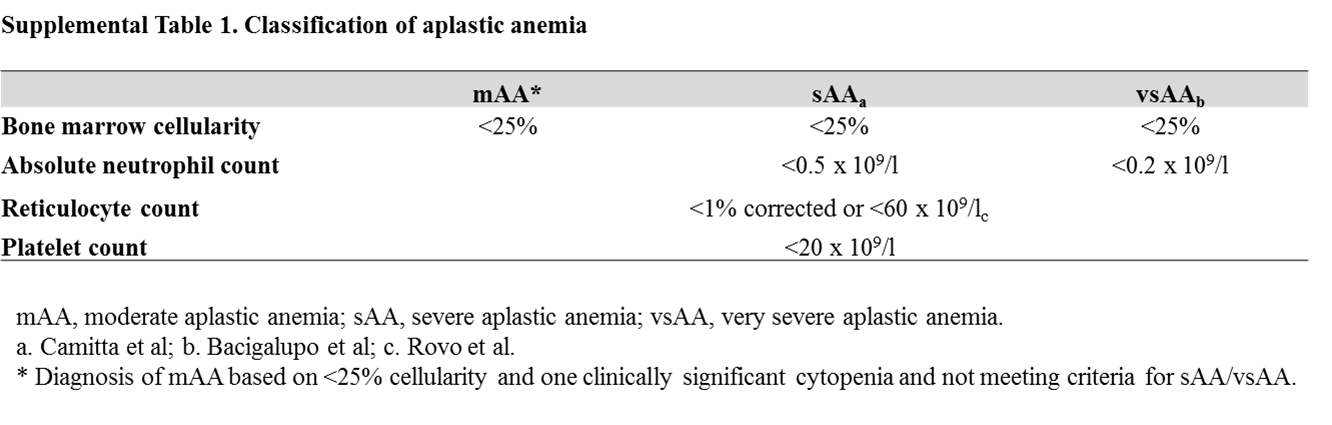
**

**
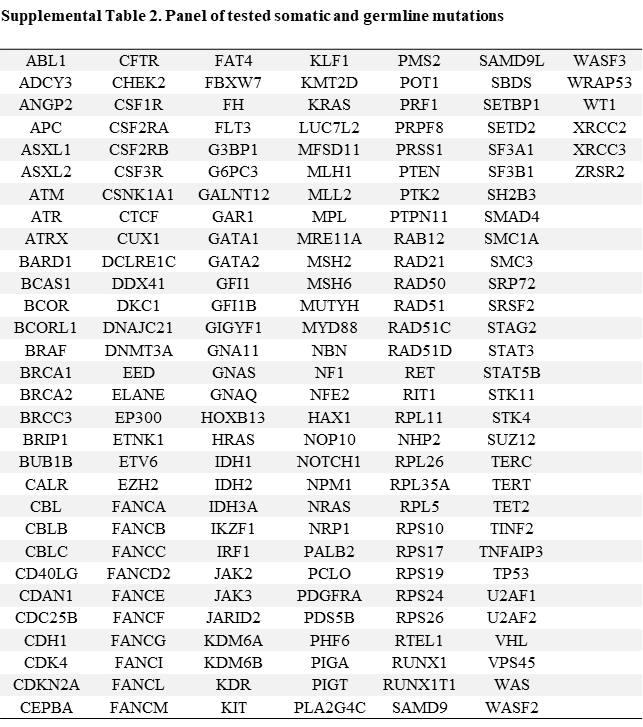
**


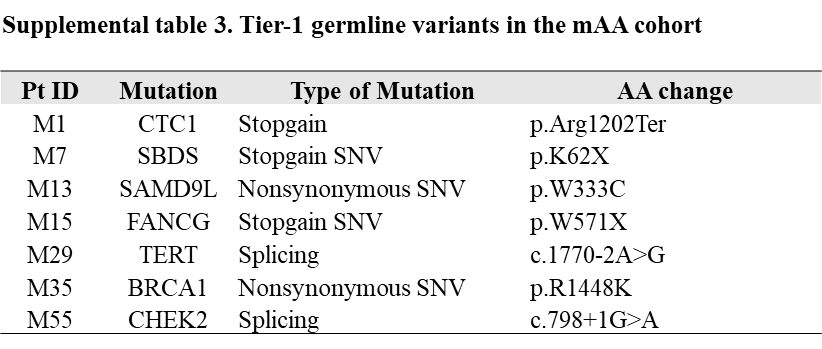


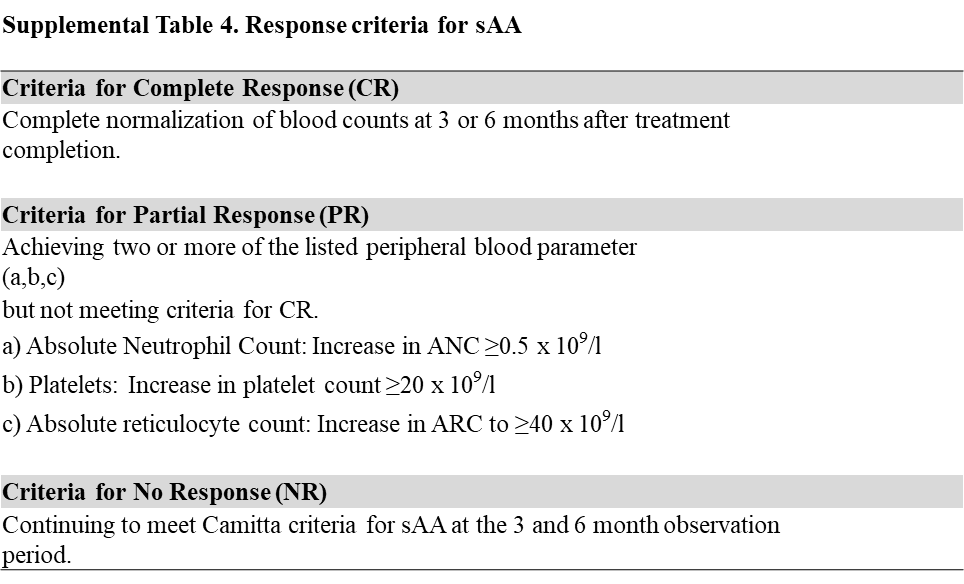


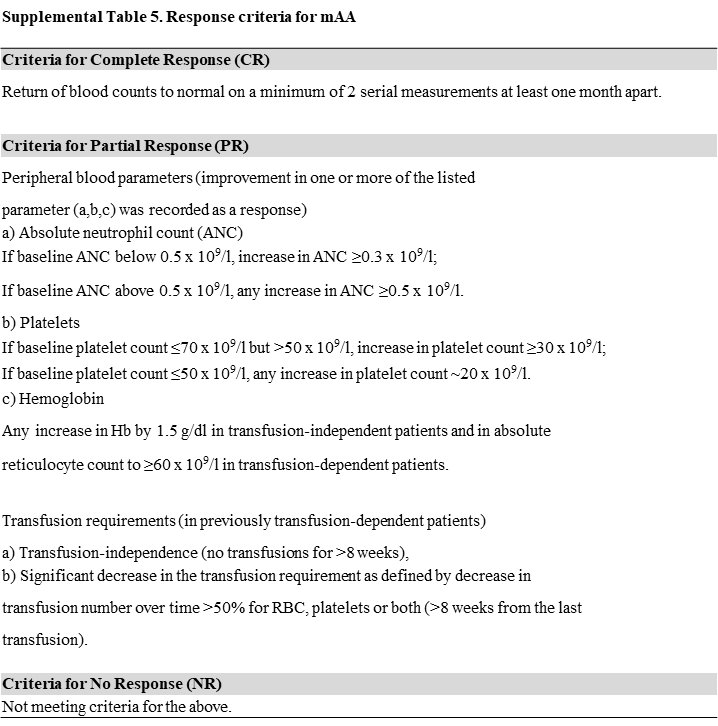

**
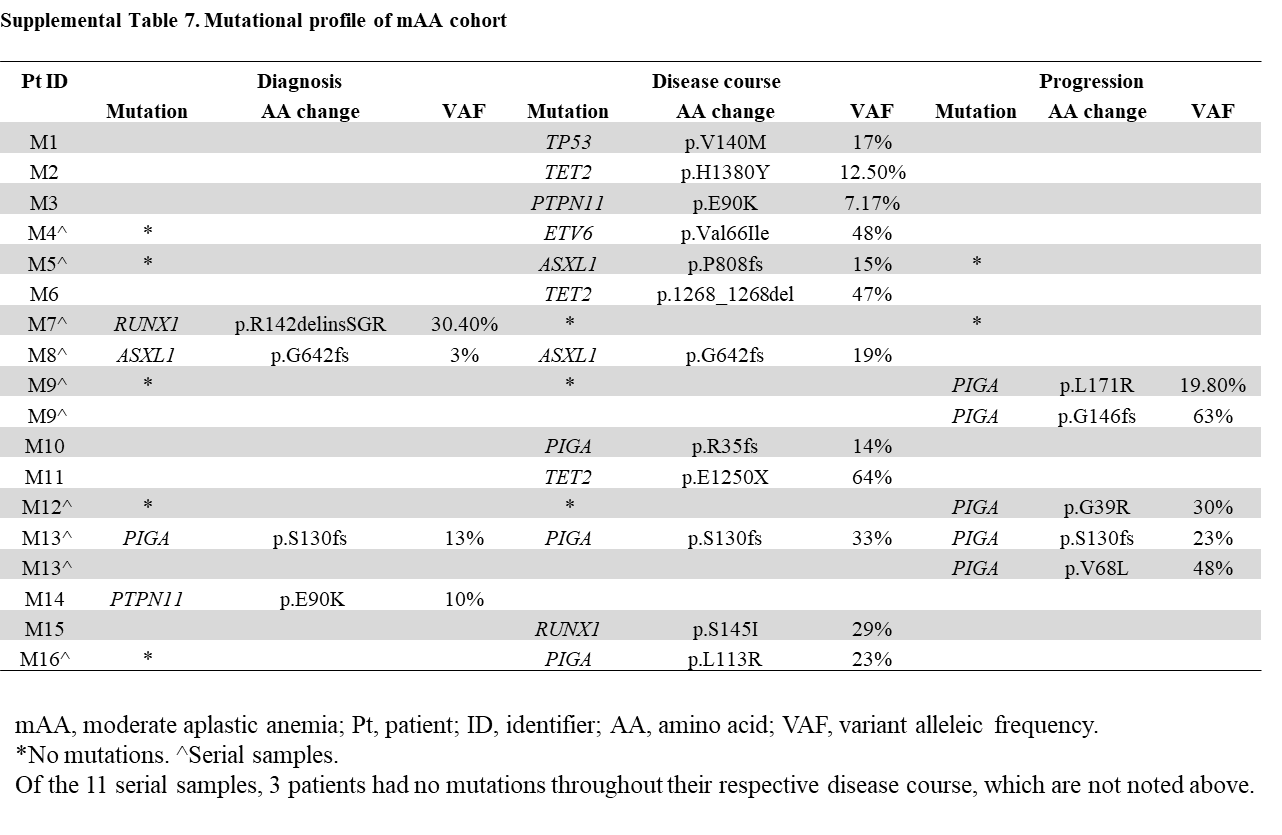
**

**
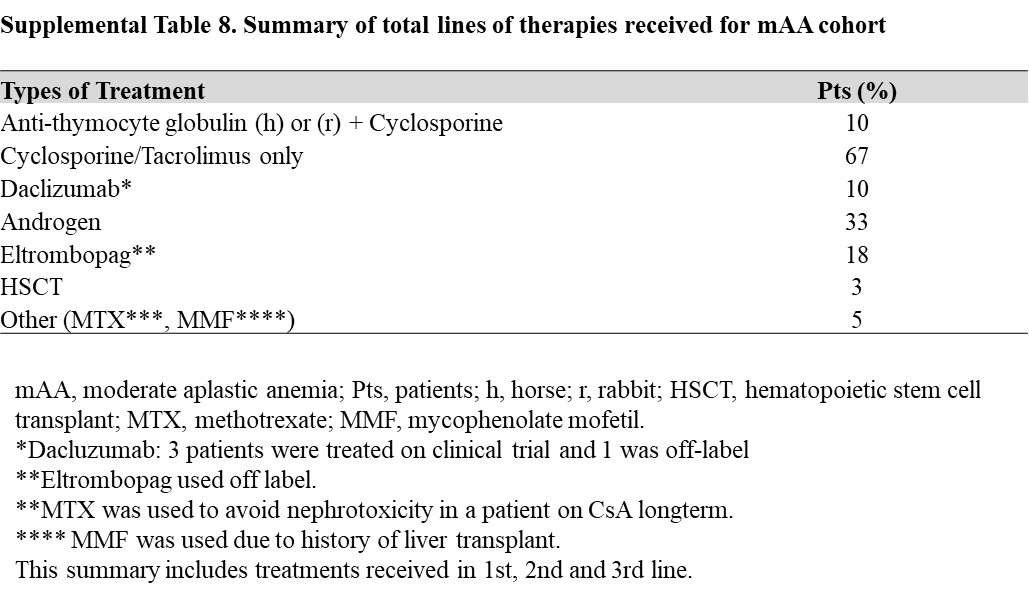
**

**
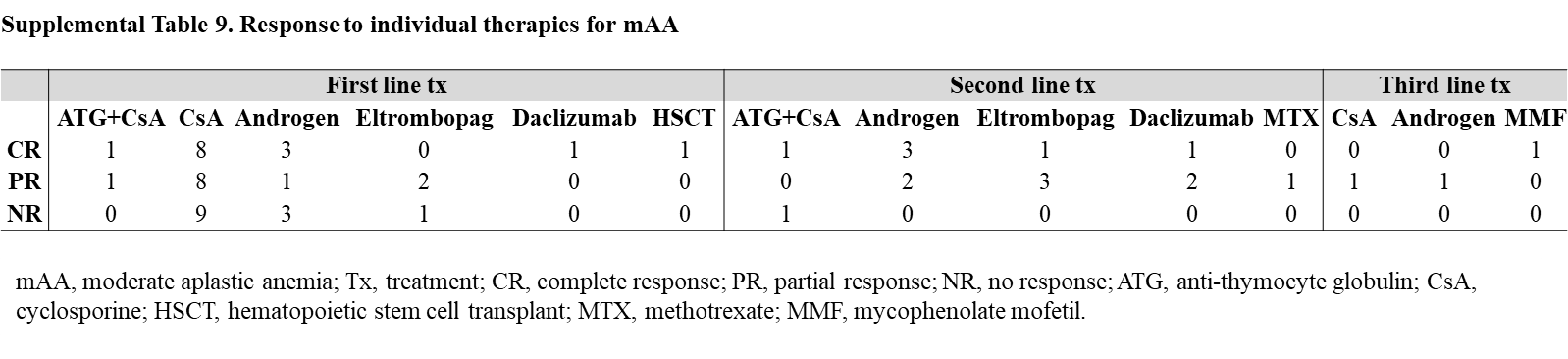
**

**3. Supplemental Figures**

**Supplemental Figure 1: Bioanalytic Algorithm for somatic variants**

**
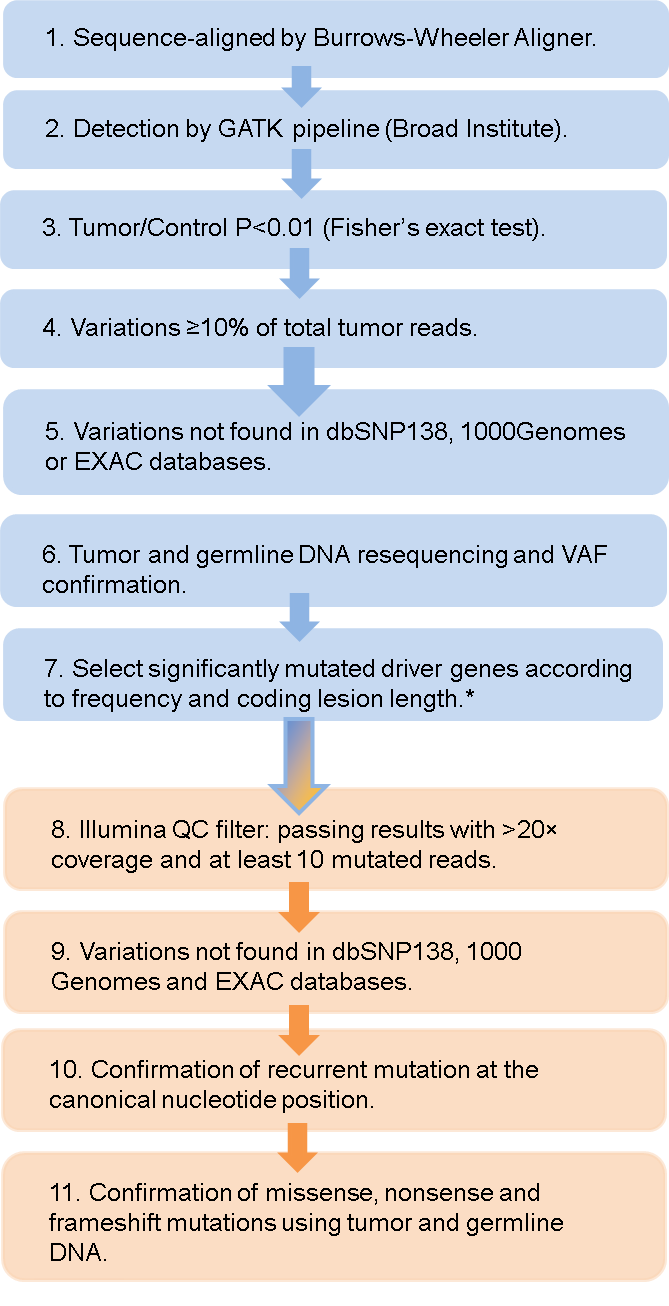
**

* Driver genes were selected as mentioned in on line method section. Multi-amplicon sequencing panel is shown in Supplementary Table 2. All available samples were subjected to targeted sequencing and all samples with adequate DNA quantity left were analyzed by SNP-array.

**Supplemental Figure 2: Bioanalytic Algorithm for germline variants**


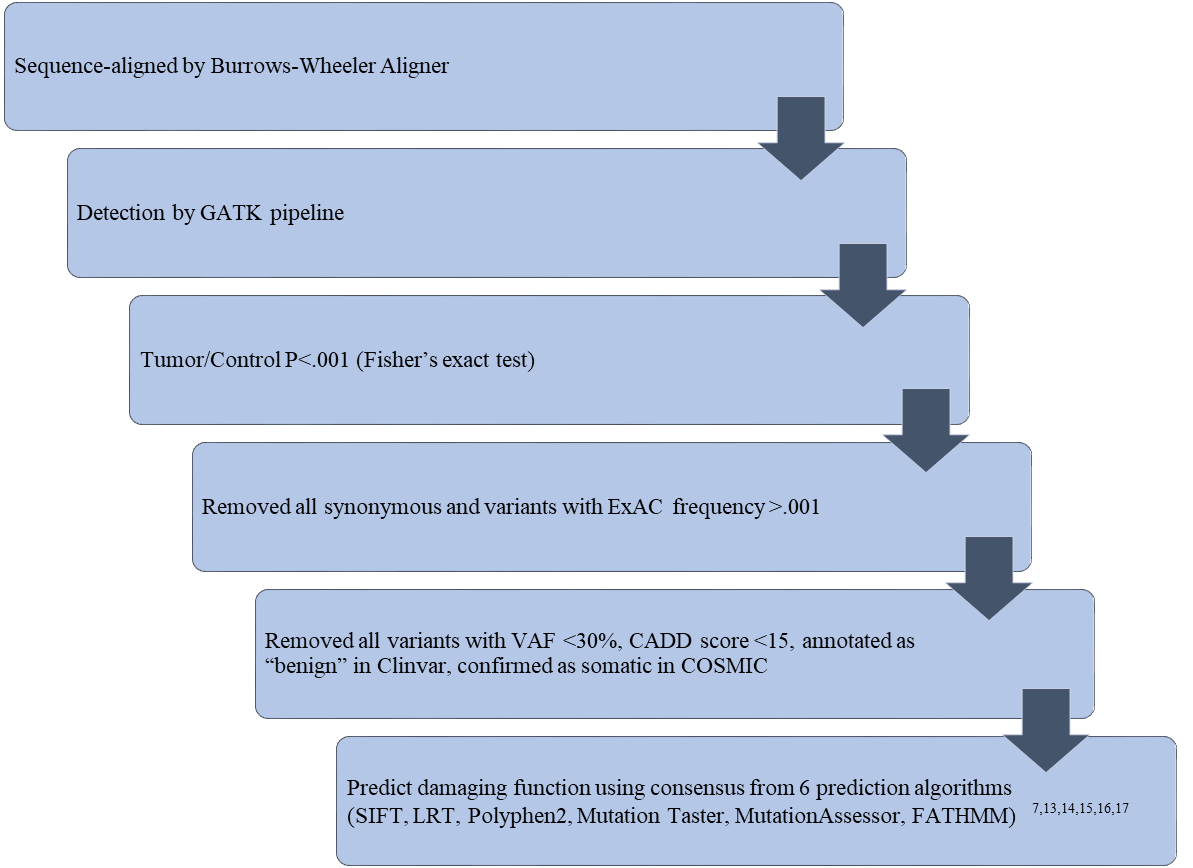


**SUPPLEMENTARY REFERENCES**

Adzhubei, I., Jordan, D.M. & Sunyaev, S.R. (2013) Predicting functional effect of human missense mutations using PolyPhen-2. *Curr Protoc Hum Genet,* **Chapter 7,** Unit7 20.

Bacigalupo, A., Hows, J., Gluckman, E., Nissen, C., Marsh, J., Van Lint, M.T., Congiu, M., De Planque, M.M., Ernst, P., McCann, S. & et al. (1988) Bone marrow transplantation (BMT) versus immunosuppression for the treatment of severe aplastic anaemia (SAA): a report of the EBMT SAA working party. *Br J Haematol,* **70,** 177-182.

Camitta, B.M., Thomas, E.D., Nathan, D.G., Santos, G., Gordon-Smith, E.C., Gale, R.P., Rappeport, J.M. & Storb, R. (1976) Severe aplastic anemia: a prospective study of the effect of early marrow transplantation on acute mortality. *Blood,* **48,** 63-70.

Chun, S. & Fay, J.C. (2009) Identification of deleterious mutations within three human genomes. *Genome Res,* **19,** 1553-1561.

Cooper, G.M., Stone, E.A., Asimenos, G., Program, N.C.S., Green, E.D., Batzoglou, S. & Sidow, A. (2005) Distribution and intensity of constraint in mammalian genomic sequence. *Genome Res,* **15,** 901-913.

Huh, J., Tiu, R.V., Gondek, L.P., O'Keefe, C.L., Jasek, M., Makishima, H., Jankowska, A.M., Jiang, Y., Verma, A., Theil, K.S., McDevitt, M.A. & Maciejewski, J.P. (2010) Characterization of chromosome arm 20q abnormalities in myeloid malignancies using genome-wide single nucleotide polymorphism array analysis. *Genes Chromosomes Cancer,* **49,** 390-399.

Kircher, M., Witten, D.M., Jain, P., O'Roak, B.J., Cooper, G.M. & Shendure, J. (2014) A general framework for estimating the relative pathogenicity of human genetic variants. *Nat Genet,* **46,** 310-315.

Kumar, P., Henikoff, S. & Ng, P.C. (2009) Predicting the effects of coding non-synonymous variants on protein function using the SIFT algorithm. *Nat Protoc,* **4,** 1073-1081.

Lek, M., Karczewski, K.J., Minikel, E.V., Samocha, K.E., Banks, E., Fennell, T., O'Donnell-Luria, A.H., Ware, J.S., Hill, A.J., Cummings, B.B., Tukiainen, T., Birnbaum, D.P., Kosmicki, J.A., Duncan, L.E., Estrada, K., Zhao, F., Zou, J., Pierce-Hoffman, E., Berghout, J., Cooper, D.N., Deflaux, N., DePristo, M., Do, R., Flannick, J., Fromer, M., Gauthier, L., Goldstein, J., Gupta, N., Howrigan, D., Kiezun, A., Kurki, M.I., Moonshine, A.L., Natarajan, P., Orozco, L., Peloso, G.M., Poplin, R., Rivas, M.A., Ruano-Rubio, V., Rose, S.A., Ruderfer, D.M., Shakir, K., Stenson, P.D., Stevens, C., Thomas, B.P., Tiao, G., Tusie-Luna, M.T., Weisburd, B., Won, H.H., Yu, D., Altshuler, D.M., Ardissino, D., Boehnke, M., Danesh, J., Donnelly, S., Elosua, R., Florez, J.C., Gabriel, S.B., Getz, G., Glatt, S.J., Hultman, C.M., Kathiresan, S., Laakso, M., McCarroll, S., McCarthy, M.I., McGovern, D., McPherson, R., Neale, B.M., Palotie, A., Purcell, S.M., Saleheen, D., Scharf, J.M., Sklar, P., Sullivan, P.F., Tuomilehto, J., Tsuang, M.T., Watkins, H.C., Wilson, J.G., Daly, M.J., MacArthur, D.G. & Exome Aggregation, C. (2016) Analysis of protein-coding genetic variation in 60,706 humans. *Nature,* **536,** 285-291.

Li, H. & Durbin, R. (2010) Fast and accurate long-read alignment with Burrows-Wheeler transform. *Bioinformatics,* **26,** 589-595.

Makishima, H., Jankowska, A.M., McDevitt, M.A., O'Keefe, C., Dujardin, S., Cazzolli, H., Przychodzen, B., Prince, C., Nicoll, J., Siddaiah, H., Shaik, M., Szpurka, H., Hsi, E., Advani, A., Paquette, R. & Maciejewski, J.P. (2011) CBL, CBLB, TET2, ASXL1, and IDH1/2 mutations and additional chromosomal aberrations constitute molecular events in chronic myelogenous leukemia. *Blood,* **117,** e198-206.

Makishima, H., Visconte, V., Sakaguchi, H., Jankowska, A.M., Abu Kar, S., Jerez, A., Przychodzen, B., Bupathi, M., Guinta, K., Afable, M.G., Sekeres, M.A., Padgett, R.A., Tiu, R.V. & Maciejewski, J.P. (2012) Mutations in the spliceosome machinery, a novel and ubiquitous pathway in leukemogenesis. *Blood,* **119,** 3203-3210.

Rovo, A., Tichelli, A., Dufour, C. & Saa-Wp, E. (2013) Diagnosis of acquired aplastic anemia. *Bone Marrow Transplant,* **48,** 162-167.

Schwarz, J.M., Rodelsperger, C., Schuelke, M. & Seelow, D. (2010) MutationTaster evaluates disease-causing potential of sequence alterations. *Nat Methods,* **7,** 575-576.

Tiu, R.V., Gondek, L.P., O'Keefe, C.L., Huh, J., Sekeres, M.A., Elson, P., McDevitt, M.A., Wang, X.F., Levis, M.J., Karp, J.E., Advani, A.S. & Maciejewski, J.P. (2009) New lesions detected by single nucleotide polymorphism array-based chromosomal analysis have important clinical impact in acute myeloid leukemia. *J Clin Oncol,* **27,** 5219-5226.

Van der Auwera, G.A., Carneiro, M.O., Hartl, C., Poplin, R., Del Angel, G., Levy-Moonshine, A., Jordan, T., Shakir, K., Roazen, D., Thibault, J., Banks, E., Garimella, K.V., Altshuler, D., Gabriel, S. & DePristo, M.A. (2013) From FastQ data to high confidence variant calls: the Genome Analysis Toolkit best practices pipeline. *Curr Protoc Bioinformatics,* **43,** 11 10 11-33.

Wang, K., Li, M. & Hakonarson, H. (2010) ANNOVAR: functional annotation of genetic variants from high-throughput sequencing data. *Nucleic Acids Res,* **38,** e164.

(Adzhubei*, et al* 2013, Bacigalupo*, et al* 1988, Camitta*, et al* 1976, Chun and Fay 2009, Cooper*, et al* 2005, Kumar*, et al* 2009, Rovo*, et al* 2013, Schwarz*, et al* 2010)
